# Ginsenoside Rg3 Reduces Lipid Accumulation with AMP-Activated Protein Kinase (AMPK) Activation in HepG2 Cells

**DOI:** 10.3390/ijms13055729

**Published:** 2012-05-11

**Authors:** Seohyun Lee, Mak-Soon Lee, Chong-Tai Kim, In-Hwan Kim, Yangha Kim

**Affiliations:** 1 Department of Nutritional Science and Food Management, Ewha Womans University, Seoul 120-750, Korea; E-Mails: hayeeun@empas.com (S.L.); troph@hanmail.net (M.-S.L.); 2 Food Bio-Nano Research Group, Korea Food Research Institute, Seongnam, Gyeonggi 463-746, Korea; E-Mail: ctkim@kfri.re.kr; 3 Department of Food and Nutrition, College of Health Sciences, Korea University, Seoul 136-703, Korea; E-Mail: k610in@korea.ac.kr

**Keywords:** Cardiovascular disease (CVD), ginsenoside Rg3, cholesterol, triglyceride, SREBP-2, HMGCR, AMPK

## Abstract

Cardiovascular disease (CVD) is one of the main causes of mortality worldwide, and dyslipidemia is a major risk factor for CVD. Ginseng has been widely used in the clinic to treat CVD. Ginsenoside Rg3, one of the major active components of ginseng, has been reported to exhibit antiobesity, antidiabetic, and cardioprotective effects. However, the effect of ginsenoside Rg3 on hepatic lipid metabolism remains unclear. Therefore, we investigated whether ginsenoside Rg3 would regulate hepatic lipid metabolism with AMP-activated protein kinase (AMPK) activation in HepG2 cells. Ginsenoside Rg3 significantly reduced hepatic cholesterol and triglyceride levels. Furthermore, ginsenoside Rg3 inhibited expression of sterol regulatory element binding protein-2 (SREBP-2) and 3-hydroxy-3-methyl glutaryl coenzyme A reductase (HMGCR). Ginsenoside Rg3 increased activity of AMPK, a major regulator of energy metabolism. These results suggest that ginsenoside Rg3 reduces hepatic lipid accumulation with inhibition of SREBP-2 and HMGCR expression and stimulation of AMPK activity in HepG2 cells. Therefore, ginsenoside Rg3 may be beneficial as a food ingredient to lower the risk of CVD by regulating dyslipidemia.

## 1. Introduction

Cardiovascular disease (CVD) is one of the major causes of mortality globally, and the mortality from CVD has been increasing at an alarming rate. The World Health Organization estimates that deaths due to CVD will rise to 23.6 million by 2030, which it is an approximate 40% increase from 2004 rates [1,2]. Dyslipidemia, including high concentrations of total cholesterol and triglycerides, is a high risk factor for CVD [1]. In the field of food science, studies on CVD concerned with dyslipidemia have focused on food ingredients that have the potential to reduce and regulate cholesterol and triglyceride concentrations.

Ginseng is one of the most well-known herbal medicines in the world [3]. Ginsenosides, the major pharmacologically-active components of ginseng, are known for their biological activities and can be divided into several different compounds, such as Rg3, Rb2, Rc, and Rg1 [3]. Among ginsenosides, ginsenoside Rg3 exhibits antiobesity [4], antidiabetic [5,6], anticancer, antihypertensive, and cardioprotective effects [7]. Ginsam, which is enriched in the ginsenoside Rg3, shows weight-lowering effects in an obese insulin-resistant animal model by changing the expression of genes involved in glucose and fatty acid metabolism [8]. Treatment with ginsenoside Rg3 is very effective for inhibiting lipid accumulation in 3T3-L1 adipocytes compared to that of Rk1 or Rg5 [9]. Nevertheless, it remains unresolved how ginsenoside Rg3 inhibits hepatic lipid accumulation and whether it regulates the expression of sterol regulatory element binding protein-2 (SREBP-2) and activation of AMP-activated protein kinase (AMPK) in HepG2 cells.

SREBP-2 plays an important role in the regulation of cholesterol homeostasis by binding and activating the promoters of SREBP-2-regulated genes, such as low-density lipoprotein receptor (LDL-R), 3′-hydroxylmethyl glutaryl coenzyme A synthase (HMGCS) and 3′-hydroxylmethyl glutaryl coenzyme A reductase (HMGCR) [10]. LDL-R is primarily responsible for the uptake of LDL from the circulation. In contrast, HMGCS and HMGCR are major enzymes in hepatic cholesterol biosynthesis [11]. Thus, SREBP-2 activation stimulates cholesterol uptake into the cells and cholesterol biosynthesis via up-regulation of LDL-R and HMGCR gene expression.

AMPK is a key sensor in the regulation of glucose and lipid metabolism. AMPK is found in all eukaryotes and is activated by an increase in the cellular AMP:ATP ratio after ATP depletion [12]. Once activated, AMPK switches off anabolic pathways such as cholesterol, fatty acid, and triglyceride biosynthesis [13]. In the liver, AMPK inactivates key lipid biosynthesis enzymes such as acetyl-CoA carboxylase-α (ACC-α) and HMGCR, which regulate fatty acid and cholesterol biosynthesis, respectively [14]. Ginseng extract increases adiponectin protein expression in 3T3-L1 adipocytes [5]. The finding that adiponectin activates AMPK and reduces cholesterol synthesis in ApoE-deficient mice suggests that the regulation of HMGCR via AMPK may be critical for regulating cholesterol metabolism *in vivo* [14]. Therefore, it is logical to hypothesize that AMPK may be involved in the ginsenoside Rg3-induced hypolipidemic effects by reducing triglyceride and cholesterol levels. This study was designed to investigate the hypolipidemic effects of ginsenoside Rg3 in HepG2 cells. We hypothesized that treatment with ginsenoside Rg3 inhibits cholesterol and triglyceride accumulation with stimulation of AMPK activity and inhibition of SREBP-2 expression in HepG2 cells.

## 2. Results and Discussion

### 2.1. Effect of Ginsenoside Rg3 on HepG2 Cell Viability

**Figure 1:**
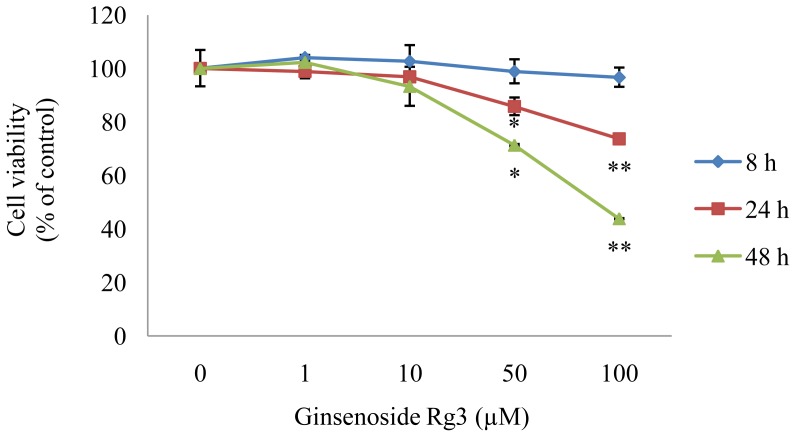
Effect of ginsenoside Rg3 on HepG2 cell viability. Cells were treated with 0 (control), 1, 10, 50, or 100 μM ginsenoside Rg3, and incubated for 8, 24, or 48 h. Cell viability was determined using the WST-8 assay. Values are expressed as mean ± SE (*n* = 3) of three independent experiments. ^*^ *P* < 0.05 and ^**^ *P* < 0.01 *versus* control treatment.

HepG2 cells were used to confirm that ginsenoside Rg3 has inhibitory effects on lipid accumulation. Cells were cultured in various concentrations of Rg3 for 8, 24, and 48 h to determine whether ginsenoside Rg3 affects HepG2 cell viability. The survival rates of HepG2 cells were unaffected in 1 or 10 μM Rg3 after 48 h incubation (Figure 1) (*P* < 0.05). In contrast, high doses (50–100 μM) of Rg3 decreased viability by 25–60% after 48 h of incubation. Thus, the nontoxic concentrations were 1–10 μM ginsenoside Rg3 in HepG2 cells.

Meticulous care is required when treating cells with ginsenoside Rg3, because cytotoxicity has been reported in several *in vitro* studies. Ginsenoside Rg3 exhibits IC_50_ (50% growth inhibition concentration) values of 41 μM in SK-HEP-1 hepatoma cancer cells [15] and potent cytotoxicity against HepG2 cells, with an IC_50_ value of approximately 50 μM after a 36 h incubation [16]. A recent study reported that HepG2 cell viability decreased following treatment with 50, 100, or 200 μg/mL Rg3 for 24 h [17]. However, cell viability remained at 90–100% in Rg3 concentrations below 10 μM in the present study. Thus, a ginsenoside Rg3 concentration of 10 μM was used in subsequent experiments.

### 2.2. Effects of Ginsenoside Rg3 on Lipid Metabolism

**Figure 2:**
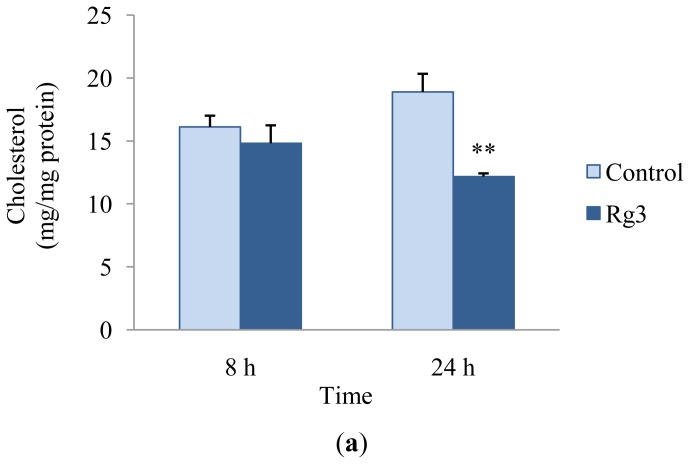

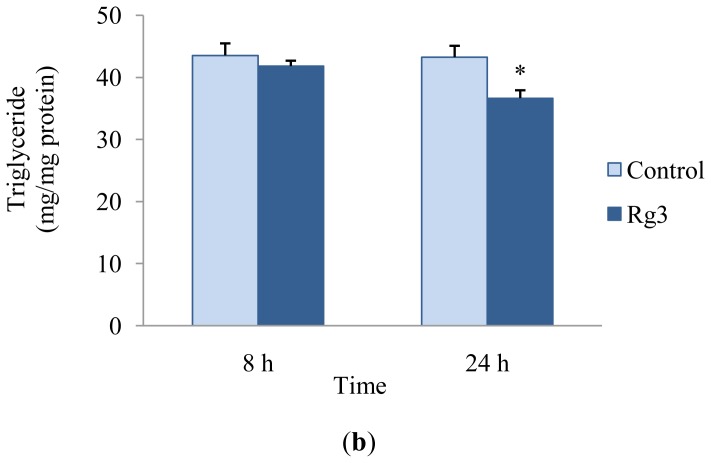
Effects of ginsenoside Rg3 on cholesterol (**a**) and triglyceride (**b**) contents in HepG2 cells. Cells were incubated with 10 μM ginsenoside Rg3 for 8 or 24 h. The intracellular cholesterol and triglyceride contents were determined by enzymatic methods. Values are expressed as mean ± SE (*n* = 3) of three independent experiments. ^*^ *P* < 0.05 and ^**^ *P* < 0.01 *versus* control treatment.

We investigated intracellular cholesterol and triglyceride contents to evaluate the effects of ginsenoside Rg3 on the reduction of cholesterol and triglyceride. HepG2 cells were treated with 10 μM ginsenoside Rg3 for 8 or 24 h. The amount of cholesterol in HepG2 cells in the presence of ginsenoside Rg3 was significantly reduced by 36% in comparison with the control after a 24 h incubation (Figure 2a). The HepG2 intracellular triglyceride content in the presence of ginsenoside Rg3 also decreased by 15% compared with that of HepG2 cells in the absence of ginsenoside Rg3 (Figure 2b). Previous studies reported that treatment with high concentrations of ginsenoside Rg3 (40–100 μM) reduce intracellular triglyceride accumulation in 3T3-L1 adipocytes [4,9]. Hwang *et al.* suggested that ginsenoside Rg3 is effective in inhibiting lipid biosynthesis by up-regulating AMPK [4]. Treatment with ginsenoside Rg3 results in a decrease in blood cholesterol and triglyceride levels in hyperlipidemic mice [5] and in obese type 2 diabetic rats [18]. Treatment with Korean red ginseng extract, which is enriched with ginsenoside Rg3, reduces the serum total cholesterol level in C57BL/6J mice fed a high-fat diet by down-regulating lipid-metabolism associated genes such as cholesterol 7α-hydroxylase (CYP7A1), which catalyzes the synthesis of cholesterol, steroids, and other lipids [19]. In this study, ginsenoside Rg3 reduced cholesterol and triglyceride accumulation in HepG2 cells.

### 2.3. Effects of Ginsenoside Rg3 on Gene Expression of SREBP-2 and HMGCR

Cellular cholesterol and fatty acid homeostasis is regulated by three members of the SREBP family, namely SREBP-1a, SREBP-1c, and SREBP-2. SREBP-2 primarily regulates cholesterol metabolism by activating genes required for cholesterol biosynthesis such as HMGCS, HMGCR, farnesyl diphosphate synthase, and squalene synthase [20]. Nascent SREBP-2 is synthesized as an inactive precursor protein bound to the endoplasmic reticulum membrane [10]. When cells are deprived of sterols, a two-step proteolytic process releases the SREBP-2 NH_2_-terminal fragment as a mature form of SREBP-2, and it binds to sterol regulatory elements in the promoter regions of genes involved in cholesterol biosynthesis [21]. A previous *in vivo* study reported that mRNA levels of all SREBP-2 target genes, such as LDL-R, HMGCS, and HMGCR, decrease with a decrease in SREBP-2 mRNA level in SREBP cleavage-activating protein-deficient mice [22].

Food ingredients, such as curcumin [11], skim milk, casein, whey, leucine, and valine [23], interfere with SREBP-2 mRNA expression, leading to a reduction in the cellular cholesterol level. These previous studies indicate that inhibiting SREBP-2 expression may be involved in the decrease of cellular cholesterol level. However, the regulatory effects of ginsenoside Rg3 on SREBP-2 and HMGCR gene expression involved in lipid metabolism have not been reported in HepG2 cells.

**Figure 3:**
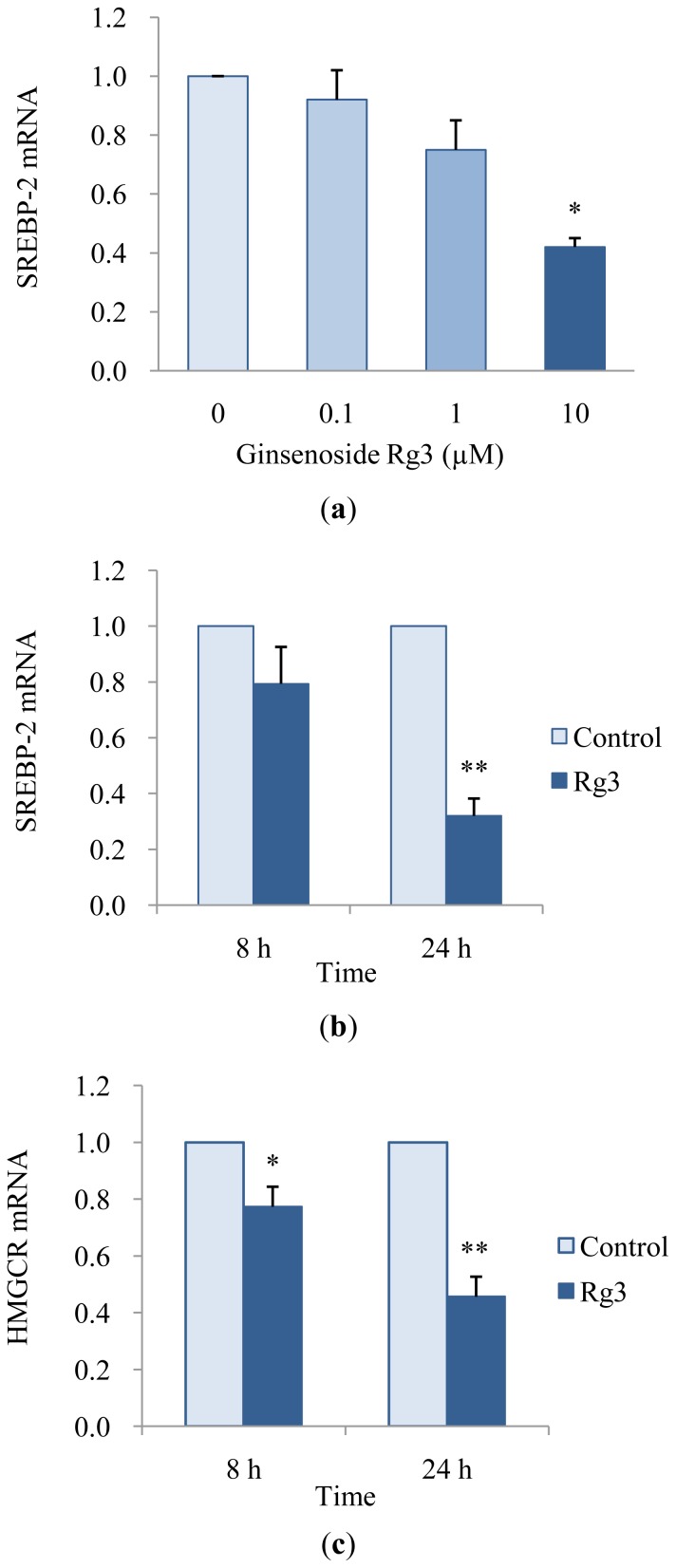
Effects of ginsenoside Rg3 on SREBP-2 (**a**), (**b**) and HMGCR (**c**) expression in HepG2 cells. Cells were treated with different concentrations (0, 0.1, 0 or 10 μM) of ginsenoside Rg3 for 24 h and were treated with 10 μM of ginsenoside Rg3 for 8 or 24 h. The mRNA of SREBP-2 and HMGCR were determined by quantitative real-time PCR. Values are expressed as mean ± SE (*n* = 3) of three independent experiments. ^*^ *P* < 0.05 and ^**^ *P* < 0.01 *versus* control treatment.

We investigated the mRNA expression of SREBP-2 and HMGCR to understand the mechanism that underlies the hypolipidemic effect of ginsenoside Rg3 in HepG2 cells. Ginsenoside Rg3 decreased mRNA expression of SREBP-2, a major direct transcriptional regulator of genes involved in cholesterol metabolism, in dose- and time-dependent manners (Figure 3a,b). Gene expression of HMGCR, which catalyzes a rate-limiting step in cholesterol synthesis, was also suppressed in a time-dependent manner (Figure 3c). Our results indicate that ginsenoside Rg3 down-regulated SREBP-2 and HMGCR mRNA expression. Consequently, inhibiting cholesterol biosynthesis by down-regulating SREBP-2 and HMGCR mRNA expression may be evidence for the hypothesis that ginsenoside Rg3 has a hypolipidemic effect in HepG2 cells.

### 2.4. Effect of Ginsenoside Rg3 on AMPK Activation

AMPK activation regulates energy metabolism that favors the inhibition of lipogenesis by inactivating ACC-α and HMGCR, the key enzymes of fatty acid synthesis and cholesterol synthesis, respectively [14]. Inactivation of ACC-α by AMPK reduces malonyl-CoA concentration, leading to stimulation of fatty acid oxidation concomitantly with an increase in ß-oxidation [24]. Treatment with metformin, an AMPK substrate, stimulates AMPK activity, which, in turn, decreases intracellular triglyceride and cholesterol contents in HepG2 cells [13]. In the cholesterol biosynthesis pathway, AMPK inhibits the conversion of HMG-CoA to mevalonate by down-regulating HMGCR [24].

**Figure 4:**
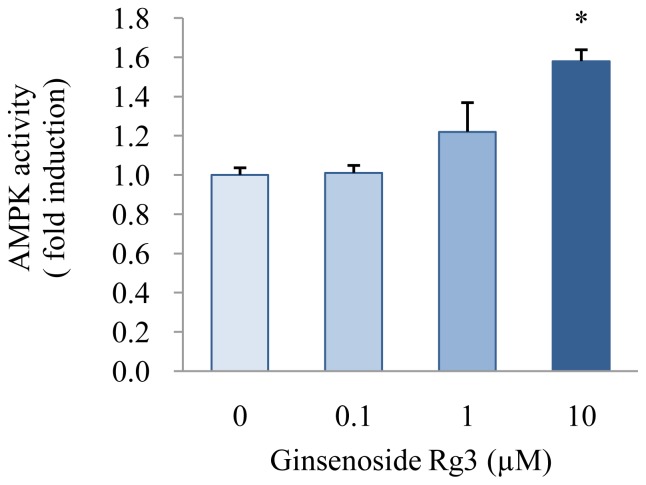
Effect of ginsenoside Rg3 on AMPK activity in HepG2 cells. Cells were treated with various concentrations (0, 0.1, 1, or 10 μM) of ginsenoside Rg3 for 24 h. AMPK activity was determined using an AMPK Kinase Assay Kit. Values are expressed as mean ± SE (*n* = 3) of three independent experiments. ^*^ *P* < 0.05 *versus* control treatment.

We treated cells with ginsenoside Rg3 to investigate whether it affects AMPK activity in HepG2 cells. As a result, AMPK activity increased by 1.22- and 1.58-fold in HepG2 cells treated with 1 and 10 μM ginsenoside Rg3, respectively, compared with that in control cells (*P* < 0.05) (Figure 4). It has been reported that AMPK activation is involved in the reduction of triglyceride accumulation by Rg3 during 3T3-L1 adipocyte differentiation [4]. Similarly, a previous *in vivo* study showed that treatment with ginsam, which is a component of ginseng produced by vinegar extraction and enriched with ginsenoside Rg3, reduces plasma total cholesterol concentration by increasing AMPK activity in obese insulin-resistant rats [8]. Our results suggest that ginsenoside Rg3 may inhibit accumulation of triglyceride and cholesterol with stimulating AMPK activity.

A recent study demonstrated that a synthetic polyphenol, S17834, stimulates AMPK activity, inhibits SREBP-2 gene expression, and reduces expression of its own target genes, such as HMGCS and HMGCR, then down-regulates cholesterol biosynthesis in the liver [25]. Although it was found that AMPK activation and down-regulation of SREBP-2 were involved in the hypolipidemic effects exerted by the Rg3, no direct correlation was presented between the AMPK and SREBP-2 signaling pathways. Therefore, a possible connection between these two effects by ginsenoside Rg3 should be investigated in further studies.

Our results suggest that the hypolipidemic effects of ginsenoside Rg3 may be caused, at least in part, by modulating the expression of SREBP-2 and AMPK activation. To our knowledge, the present study is the first to suggest that ginsenoside Rg3 plays a role as a regulator of the expression of SREBP-2 and AMPK activation involved in lipid metabolism in HepG2 cells.

## 3. Experimental Section

### 3.1. Reagents

Ginsenoside Rg3 (purity >98%) was purchased from Santa Cruz Biotechnology (Santa Cruz, CA, USA). The human HepG2 cell line was obtained from American Type Culture Collection (Manassas, VA, USA). Dulbecco’s modified Eagle’s medium (DMEM), phosphate-buffered saline, pH 7.4 (PBS), fetal bovine serum (FBS), glutamine, and penicillin-streptomycin, TRIzol reagent, and M-MLV reverse transcriptase were purchased from Gibco/Invitrogen (Grand Island, NY, USA). The Universal SYBR Green PCR Master Mix was obtained from Qiagen (Valencia, CA, USA). A cell count kit-8 (CCK-8) was purchased from Dojindo Laboratories (Kumamoto, Japan). Assay kits for cholesterol and triglyceride were obtained from Asan Pharmaceutical Co. (Seoul, Korea). The AMPK Kinase Assay kit was purchased from Cyclex (Nagano, Japan). A bicinchoninic acid (BCA) protein assay kit was obtained from Thermo Scientific (Pittsburgh, PA, USA). Dimethyl sulfoxide (DMSO) and Triton X-100 were purchased from Sigma-Aldrich (St. Louis, MO, USA).

### 3.2. Cell Culture

Human HepG2 cells were cultured in DMEM, supplemented with 10% (v/v) FBS and penicillin-streptomycin (100 units/mL) at 37 °C and 5% CO_2_. Ginsenoside Rg3 was dissolved in DMSO. The final DMSO concentration in culture medium was 0.01%. Cells were treated with different concentrations of ginsenoside Rg3 in serum-free media for 8 and 24 h. Control cells were treated with 0.01% DMSO without ginsenoside Rg3. All measurements were performed in triplicate for each treatment.

### 3.3. Cytotoxicity Assay

Cell viability was determined by the WST-8 [2-(2-methoxy-4-nitropheyl)-3-(4-nitrophenyl)-5-(2,4-dinitrophenyl)-2H-tetrazolium, monosodium salt] assay, using a CCK-8 kit according to the manufacturer’s instructions. The assay is based on the cleavage of the WST-8 tetrazolium salt to formazan by cellular mitochondrial dehydrogenase. Cell viability was determined following culture in 96-well plates at a seeding density of 10^4^ cells/well. Cells were treated with 0, 1, 10, 50, or 100 μM of ginsenoside Rg3 for 8, 24, and 48 h at 37 °C. WST-8/1-methoxy-phenazine methosulfate solution was added to each well and incubated for 3 h at 37 °C. The absorbance at 450 nm was measured using a Varioskan plate reader (Thermo Electron, Waltham, MA, USA). Values are expressed as a percentage of control cells without ginsenoside Rg3 supplementation.

### 3.4. Cholesterol and Triglyceride Assay

Cells were lysed in a lysis buffer consisting of 1% Triton X-100 in PBS, and the cellular cholesterol and triglyceride were measured using enzymatic colorimetric assay kits. The cellular protein concentration was determined using a BCA protein assay kit. Cellular cholesterol and triglyceride were normalized to cellular protein content.

### 3.5. Quantitative Real-Time Polymerase Chain Reaction (PCR)

Total RNA was extracted from HepG2 cells using TRIzol reagent. Complimentary DNAs were synthesized from 4 μg of RNA using M-MLV reverse transcriptase. After cDNA synthesis, quantitative real-time PCR was performed in 20 μL of Universal SYBR Green PCR Master Mix using a fluorometric thermal cycler (Corbett Research, Mortlake, NSW, Australia). Primers were designed using an on-line program (primer3_http://www.cgivo.2) [26]. The sequences of the sense and antisense primers used for amplification were as follows: SREBP-2, 5′-GTGGGACCATTCTGACCACA-3′ and 5′-GTTGTCCGCCTTTCTCCTTC-3′; HMGCR, 5′-ACTTATGGCAGCATTGGCAG-3′ and 5′-ACTG TCGGGCTATTCAGGCT-3′; β-actin, 5′-GGACCTGACTGACTACCTCA-3′ and 5′-GCACAGCTTC TCCTTAATGT-3′. The delta C_t_ method was used for relative quantification [27]. The delta C_t_ value for each sample was determined by calculating the difference between the C_t_ value of the target gene and the C_t_ value of the β-actin reference gene. The normalized level of expression of the target gene in each sample was calculated using the formula 2^−ΔΔCt^. Values were expressed as fold change over the control.

### 3.6. AMPK Activity Assay

AMPK activity was analyzed using an AMPK Kinase Assay kit according to the manufacturer’s instructions. Briefly, samples were incubated for 30 min at 30 °C in a precoated plate with a substrate peptide that corresponded to mouse insulin receptor substrate-1 (IRS-1). AMPK activity was measured by monitoring the phosphorylation of Ser 789 on IRS-1 using an anti-mouse phospho-Ser 789 IRS-1 monoclonal antibody and peroxidase-coupled anti-mouse IgG. Conversion of the chromogenic substrate tetramethylbenzidine was quantified by measuring absorbance at 450 nm. Protein was determined using a BCA protein assay kit. Values for AMPK activity were expressed as a fold increase over the control.

### 3.7. Statistical Analysis

Values are expressed as means ± standard errors (SE). Statistical analyses were performed using SPSS software version 19 (Chicago, IL, USA). Significant differences among treatment groups were analyzed using an unpaired Student's two-tailed *t* test and a one-way analysis of variance followed by *post hoc* Tukey’s multiple comparison tests. *P* < 0.05 was taken to indicate a significant difference.

## 4. Conclusions

We demonstrated that ginsenoside Rg3 inhibited cholesterol biosynthesis and triglyceride accumulation in HepG2 cells. Treatment with ginsenoside Rg3 resulted in reduced cholesterol and triglyceride levels in HepG2 cells. This study provides biochemical and genetic evidences that the effects of ginsenoside Rg3 on the decrease of hepatic cholesterol and triglyceride levels depend on the regulation of SREBP-2 and AMPK. Accordingly, ginsenoside Rg3 has great potential as a bioactive food ingredient for the prevention of CVD by regulating dyslipidemia.
